# Speaking Pictures, Silent Voices: Female Athletes and the Negotiation of Selfhood

**DOI:** 10.1007/s12124-020-09577-6

**Published:** 2020-10-07

**Authors:** Hannah Intezar

**Affiliations:** University of Bradford, Richmond Rd, Bradford, BD7 1DP UK

**Keywords:** Female athletes, Selfhood, Identity, The body, Bakhtin

## Abstract

Combining Mikhail Bakhtin’s (1990) theoretical position on Architectonics and Erving Goffman’s (1979) writings on visual content analysis, the aim of this paper is to explore how female athletes are caught in a complex matrix of power, post – feminist neoliberalism, and self – presentation. The visual images they choose to portray are, therefore, perfect for determining how this cohort of women negotiates social discourses around identity and femininity. Appropriating the Bakhtinian notion of architectonic unity, not only provides an alternative theoretical lens for enquiries concerning the body, identity, and selfhood, but also initiates some thought provoking questions around neoliberal feminism and ‘new femininity.’ This paper advances on previous research by exemplifying how Serena Williams (considered the greatest female tennis player of all time) combines both her femininity and strong physicality to self – shape a myth – like persona, setting her apart from traditional stereotypes of femininity and ‘femaleness.’

## Introduction

This paper explores the grey area occupied by female athletes, in terms of media self – representation and their display of *romanticised* physical strength in visual form. It argues for the ontological dangers of such self – marketing. More specifically, when such forms of self – shaping lead to a lack of selfhood coherence, a ‘sense of the [Architectonic] whole’ is missing. In, Joseph Williams’s, terms coherence is: ‘seeing what all the sentences in a piece of writing add up to, the way all the pieces in a puzzle add up to the picture on the box’ (Williams and Colomb 2010: 71). Ontological friction arises when the picture on the box, so to speak, does not correspond with the puzzle pieces within. Small pockets of sports media audiences, for instance, may be comfortable with viewing aesthetically strong female athletes, hence, pushing the boundaries of ideological restrictions surrounding ‘femininity.’ This progressive attitude, however, is limited and outweighed by derogatory and body shaming rhetoric widely used on social media (Ingle 2014; Carter-Francique and Richardson 2016). To exemplify this, I draw on female athletes, specifically Serena Williams (female tennis player), as a case study because they are the most visible cohort of women who challenge traditional modes of feminineness by displaying prowess.

The primary reason for focusing on tennis is that between 2012 and 2014 the Women’s Tennis Association (WTA) launched their campaign entitled, ‘Strong is Beautiful.’ This was one of the most comprehensive and explicit activities undertaken by a women’s sports association to address the subject of female athletes and body positivity. There are also several reasons for focusing on Williams. Since giving birth, Williams has explicitly begun to challenge social boundaries around the athletic female body, both pictorially and rhetorically. The focal point, therefore, is Williams’s visual representation and the re – shaping of ideologies surrounding her body, both on and off the court. She explicitly identifies as a feminist hoping to break barriers: ‘I want women to know that it’s okay … That you can be whatever size you are and you can be beautiful inside and out. We’re always told what’s beautiful, and what’s not, and that’s not right’ (Haskell 2018: 110). Through recent scholarship on neoliberal feminism, female athletes and ‘new femininity’, we know that, for some, feminist identity has not only been re – shaped as an individual endeavour, but is also firmly located within The Market (Gill and Scharff 2011; Gwynne 2013; Moran 2017; Toffoletti and Thorpe 2018a). The influence of women in market economy has initiated post – feminist rhetoric, which commodifies feminism through figures of individual empowered female market consumers (Tasker and Negra 2007). This raises two reflexive questions. First, if feminism has indeed become a solitary activity and is no longer perceived as a collective sisterhood, what impact does this alternative narrative of feminism have on the advocator’s identity and selfhood. Second, for post – feminism’s claims of individual empowerment and agency to function successfully, prerequisites the denial of *all* external forces. Any alternatives are viewed as implying victimhood (Gill and Scharff 2011). The following section delineates how such ‘blind – spots’ obscures the subjugating discourses around what entails marketability, and its ontological impact.

Although post – feminist reflections on aforementioned issues are somewhat murky and ambivalent (Konjer et al. 2019), considering the negative response lanced towards public figures advancing feminist agendas, such as Williams, this question is becoming increasingly important (Gill 2016; Tredway 2020; Locke and Lawthom 2018). In addition to her illustrious career, she occupies an intriguing space between hyper – visibility and invisibility (Douglas 2012). When referring to Williams, there is also another undercurrent theme to acknowledge, that of race. Scholars have gone far as considering Williams as the torchbearer of third – wave black feminism and a black cultural icon and the challenger of ‘misogynoir’ (Ifekwunigwe 2009, 2016, 2018; Bailey 2018).

### Female Athletes, Media and the Body: Moving from the Grotesque to the Classical

Much has been written around the uniformity of embodied experience within a culture (Lakoff and Johnson 1980; Johnson 2008), and its social formation. Some scholars, such as Lorber and Martin (2013), maintain that regardless of how self – alienated and internally divided one becomes, it is impossible to escape one’s bodily envelope. Informed by Bakhtin (1968) notions of The Body, this section explores the evolution of Western social discourses surrounding the body, and the positioning of the athletic female body on this complex axis.

Bakhtin (1968), for instance, offered a dichotomous concept of the body, which he labelled ‘Classical’ and ‘Grotesque.’ While the former donates an idealised, self – constrained and harmonised physical form that can be put on display, the latter is imperfect, messy, concerned with the lower – spectrum and porous boundaries. Moreover, the Grotesque body renounces the discontinuity between embodied life and its socio – historical context, known as the ‘Carnal Self’ (Bakhtin 1968). Embodiment, therefore, is regarded as a formation of cultural discourse, but this does imply that embodiment is experienced in a binary manner, rather, that it exists in *multiplicity* (Bakhtin 1968; Traversa 2012). The emergence of Modernity, however, inculcated self – disciple, body regulation (Foucault 1979), and the gendered ideologies (Philips 2018); therefore, expunging embodied experience of its multiplicity. Notions of the body transcend from unfinalisability to totalisation and uniformity, which occur because, regardless of its multiplicity, comprehension of physical functionality can only be experienced in terms of culturally available discourses (Mauss 1973). This is further complicated when one considers consumption practices and the subjecting ‘male gaze’ (Mulvey 1989), where the multiplicity of the open Grotesque female body is modified and (re) constructed into the closed Classical body. Yet, peculiarly, for marketability, still maintaining its heightened display of sexuality.

There have been feminist arguments, however, on how said sexualised female athletes have transposed such ‘hyper – sexuality’ to their financial advantage (Kim and Sagas 2014; Toffoletti and Thorpe 2018b). Indeed, not only have they carved a space for ‘economical visibility’ but have also transfigured the discourse of sexualisation from problematic to a source of empowerment (Evans et al. 2010; Hansen 2017). Commodification of the body, of course, and feminist response to it, is not limited to the field of sports. For instance, much has been debated, both academically and in the wider press, on Beyoncé (singer-songwriter) and her visual album *Lemonade*, which was marketed as a feminist creative output promoting the African – American female body and ‘sex – positivity’ (Hartmann 2017). The debates surrounding ‘sex – positivity’, however, are complex and murky. Initially, the term emerged from the turbulent period of Feminist Sex Wars (Ivanski and Kohut 2017), where different feminist positions debuted the impact of pornography on female empowerment. On the one hand, Radical feminist scholars maintained that pornography represented women as weak and disempowered victims, lacking sexual agency (Boyle 2000). On the other hand, such interpretations of the term were denounced by a ‘sex positive’ movement within feminism, and Queer Theory, that campaigned for the liberalisation of female and queer sexuality through transgressive sexual acts Showden (2012). Scholars, therefore, have employed the term in varying contexts (Glick 2000), and ‘sex – positivity’ has become paradoxical, when associated with terms such as ‘sexual commodification’ and consumption (Plummer 2012), where all forms of sexuality are negotiated as objects of capital exchange in The Market. Unsurprisingly, ‘sex – positivity’ is also associated with the feminist ‘post – pornography’ movement (Gregory and Lorange 2018), which advocated for the stigmatisation of porn and its production specifically for the *female* consumer’s gaze. Nevertheless, research focusing on audio – visual media reiterates its effectiveness in re – enforcing traditional hegemonic discourses of gender and sexuality (Tehseem et al. 2019; Rubio 2018; Ademola 2009), and therefore, the ‘male – gaze.’

Moreover, while sex – positivity and explicit sexual exploitation can foster positive self – concepts and self – determination, particularly for minority women (Parent et al. 2015), Capitalising it within The Market can result in exploitation and harm (Horley and Clarke 2016). Therefore, for some, a culture of hyper – sexuality and sex – positivity veils female oppression through subjugation of the female body: ‘I see [Hook stated] a part of Beyoncé that is in fact anti-feminist – that is a terrorist, especially in terms of the impact on young girls’ (Cocker 2014). Hence, slogans such as ‘girl – power,’ ‘can – do girls’ (Harris 2004), and ‘top – girls’ (McRobbie 2007), endorse a Classical body discourse.

Notably, the sexualised Classical body commodified by Beyoncé differs significantly from that of female athletes at the peak of their fitness. Where the body, if not entirely Grotesque in a Bakhtinian sense, certainly lacks self – control. As the analysis section posits, while athletes in ‘live action’ are dependent on openness of the Grotesque body (sweat, blood, saliva and tears), publicly they are pressured to portray Classical refinement, resulting in architectonic friction. As noted by former tennis player, Pam Shriver, ‘That is really an important acceptance for some female athletes, that their best body type, their best performance build, is one that is not thin; it’s one of power’ (cited in Rothenberg 2015). In contrast to their popular culture counterparts, therefore, female athletes walk an interesting tightrope, between embracing their strong powerful athletic bodies and displaying cultural ideals of beauty and body shape: often perpetuated by the ‘male – gaze’.

Even in post – feminist popular culture (Cobb 2011), women in motion pictures are stereotyped and sexualised (Smith et al. 2010), the difference being that subjective structures are masked behind façade of female empowerment and independence. Importantly, the difference between popular culture beauty representation and female athletes is that, for the latter, spectators *do* see beyond the curtain. There is little avenue for female athletes to retain their surplus during ‘live play’ from their audiences’ gaze. Interestingly, as televised sports is predominantly considered ‘Dude Time’ (Cooky et al. 2015), Christensen and Deutsch (2015) speculate whether the athletic physical appearance of female volleyball players is illustrious in the media because, although at the peak of physical fitness, their physicality remains conventionally ‘feminine’ (Cooky et al. 2013; Bennett et al. 2017).

Similarly, research on the media representations of female athletes has revealed the continuous maintenance of hegemonic values and male prioritisation in sports (Krane 2001; Bruce 2016). When female athletes receive media attention, feminine attributes, such as aesthetics and sexuality, are foregrounded over what is perceived as masculine qualities, such as strength and power; hence, reiterating gender differences in sports (Lisec and McDonald 2012; Barak et al. 2018). Indeed, studies exploring adolescent girls, or women, expressing interest in supposedly masculine activities, such as contact sports, has revealed that they often endure type – casting and the questioning of their sexual orientation (Carty 2005; Harrison and Secarea 2010; Daniels and Wartena 2011). To counter such stereotypes, and negative derogatory connotations, a significant number of female athletes have attempted to accentuate their femininity and their sexuality. In doing so, they appropriate themselves into heteronormative discourse (Harrison and Secarea 2010; Antunovic and Hardin 2012). In terms of tennis, when prompted to express her opinions on media and body positivity, former world No.1, Caroline Wozniacki responded that:

Right now I’m a tennis player, so I’m going to do everything I can to be the best tennis player that I can be … If that means that I need to add a little muscle to my legs or my butt or whatever, then that’s what I’m going to do. I can be a model after I finish **(**Rothenberg 2015).

Wozniacki’s dichotomisation between being an athletic ‘tennis player’ with ‘muscles’ versus ‘a model’ is particularly noteworthy. As discussed below, female tennis plays foregrounding their sexuality and femininity in pictorial media is not necessarily the point of architectonic friction. Rather, it is the continual external impositions, which become internal, to balance their athletic physicality to heteronormative conventions of femininity, or ‘femaleness.’

The Bakhtinian body encompasses continuity between embodied experience and socio – historical context (Traversa 2012), where the body exists in blurred boundaries and multiplicity, the emergence of the Classical body, however, has fortified said boundaries. The addition of heteronormative discourse has resulted in embodied multiplicity becoming subjugating, rather than agentic. As explicated below, this fragmented discourse of remaining physically and vocally ‘feminine’ seems to exist inside a given sport, as much as outside it. In order to analyse and understand how female athletes, such as Williams, risk selfhood fragmentation, I undertake a critical discourse analysis of their pictorial depictions and interview rhetoric. Whereas Fairclough (1989) draws on Bakhtin’s later work, I draw on his earlier writings on Architectonics. Therefore, bringing Bakhtin’s framework closer to new feminism concerns of *individualism* in place of collective feminism.

## Theoretical Framework

### Bakhtin and Architectonics

Mikhail Bakhtin’s theories are embedded in his understanding of everyday social discourse, an aspect he sustained throughout his career, even while he wrote about literary theory (Morson and Emerson 1990). Discourses and tropes embedded within the text, he argued, were transcendental to not only social discourse, but also offer a dialogical framework to explore the dynamics between self and other in everyday human action. It is not surprising, therefore, that Bakhtinian philosophy has been championed as an epistemological position in qualitative research (Hong et al. 2016), particularly in terms of dialogical interaction giving rise to the self (Hermans 2001, 2002; Sullivan 2007), and the construction of individual identity cultural positioning (Skinner et al. 2001).

As with many of Bakhtin’s theoretical concepts, the definition of his work on Architectonics has evolved, depending on the discipline in which it is being appropriated (Diaz-Diocaretz 1989), and the concept of Architectonics is no different. For early thinkers such as Aristotle, it concerns architecture, specifically the construction of buildings by a craftsmen who understands ‘the matter and makes the product’ (McKeon 1987: 03). In eighteenth century, however, Architectonics emerged reconceptualised as an abstract concept. Immanuel Kant (1985 originally published in 1781) distinguished between ‘technical unity’ and ‘architectonic unity,’ referred to as ‘Wholeness’ in this paper. Wholeness attained architectonically, Kant wrote, ‘originates from an idea’ (Kant 1985: 655). In contrast, Wholeness achieved without Architectonics, is where one is merely *conforming* technically to structural requirements of a given form. Wholeness attainted in such a manner is false and constrained. Architectonics, therefore, began to be understood after the late eighteenth century as architectural *or* artistic elements, which harmonised a single structure. Bakhtin’s (1990) response to the Kantian concept is as follows:

A whole is called “mechanical” when its constituent elements are united only in space and time by *some external connection and are not imbued with internal unity of meaning.* The parts of such a whole are contiguous and touch each other, *but in themselves they remain alien to each other* (Bakhtin 1990: 01, *emphasis added*).

This is akin to Bakhtin’s latter writings on the interlinked concepts of the novelistic author-hero relationship and the dialogic relations between the self and other; arguably, mediations for which, began with his work on Architectonics. Similar to the Architectonic Whole, in the hero-author dynamics, the hero is a constructive whole, which is a manifestation of the author’s response to said hero, which consummates them into a ‘unitary and unique whole that is a concrete, intuitable whole, but also a whole of meaning’ (Bakhtin 1990: 05). Importantly, comparable to female athletes, the author-hero relationship, much like the social self – other dependency, is composed of two separate consciousnesses that are in a tension filled reciprocal relationship.

Essentially, Architectonics are dialogical and evince the cultural and social capacity to co-construct meanings of individual experience, thereby having the power to alter how we experience our selfhood and identity. Developing from Williams and Colomb’s (2010) definition mentioned earlier, the Bakhtinian interpretation of Architectonic unity emphasises a greater sense of The Whole (identity of the female athlete) rather than simply the whole of the topic (female athletes and media). It involves an understanding of the individual’s personal relationship to the subject under discussion, (Serena Williams’s relationship with the media). For example, ‘as architects consider mass and the forces of gravity that push and pull on a building, architectonics as a metaphor implies the invisible social forces, primarily in language, that surround and define us’ (Greer 2013: 72). As individuals, Greer (2013) continues, we organise components of the world, that we encounter, in our own consciousness and create our own identity in relation to said items. We, therefore, construct a personal understanding of the relationship of parts to the Whole. In this sense, these Wholes are another word for meaning. When attempting to understand a given stimuli, one struggle to form Architectonic wholes as one attempts to understand and relate to it. This element of negotiating, of comprehending , and positioning oneself within the situation (Paré 2007), is an essential aspect of attaining Architectonic wholeness. Without this self-awareness, the unity formed is surface level and mechanical (Bakhtin 1990). The personal, or self – awareness, is pivotal in architectonics: ‘In contrast to traditional writing, architectonic writing requires the writer to understand her relationship with the subject and to become personally engaged when writing about it in order to compose an architectonically sound and thus effective text’ (Paré 2007: 48). Furthermore, ‘Knowledge of individual parts, portions of information without a clear relationship one to the other and to the entire text, provides no true understanding’ (Greer 2001: 58).

The analysis that follows discloses how this sense of self – awareness, of becoming a ‘true’ Architectonic whole, is increasingly receding for female athletes in an ever-expanding post – feminist, neoliberal virtual space. With the rise of social platforms and self – presentation, both visually and textually, they are struggling with attaining an *authentic self*; therefore, are in danger of conforming into *architectural mechanical* Wholes, Wholes that subscribe to heteronormative and hyper – sexual discourses of ‘womanness.’

## Methodology

### Goffman, Visual Representations and Content Analysis

The gendered double standard in advertisement and popular visual media is not unique. Recent feminist scholarship, however, has explored how female athletes prefer and choose to be visually represented in media (Smith and Sanderson 2015; Toffoletti and Thorpe 2018b; Konjer et al. 2019). For a comprehensive ‘serious engagement’ with the self – subjectification of women in the media, one ‘needs to address the extent to which the construction of active, desiring subjecthood within the verbal texts of such adverts may act as an alibi for visual representation’ (Gill 2008: 438). Cultural psychology has argued for a semiotic epistemological position to explore visual forms (Innis 2012), semiotic textual formation (Sergeevna 2018), and the multiplicity of meaning. There are two specific reasons, however, why this paper undertakes a Goffman (1979) visual content analysis, initially introduced in his work on *Gender Advertisement*. First, the crux of this project is to explore the *negotiation* of visual meaning – making between Williams and the audience, an aspect undervalued by a psycho – semiotic framework. Second, as discussed above, female athletes are caught between self – presentations of traditional femininity and their athletic self. Extending on the literary movement of Dramatism, Goffman’s (1979) work is not necessarily concerned with the economical or ‘erotic capital’ aspects of mass media visual images, rather, his drive is to explore the differences between images for public and private viewing (Konjer et al. 2019). In context of gender, he also emphasised the importance of capturing the differences between the impressions one *intends* to project deliberately, in comparison to impressions one projects *unintentionally*; through non – verbal behaviour or body language. As posited below, intentionality and body – language are key factors for Williams’s re – shaping of traditional gender conventions. Indeed, shoots for traditional magazines such as *Sports Illustrated* are now a *collaborative* process, and one assumes that social media platform accounts such as Instagram are mediated by the athlete themselves (Barak et al. 2018).

Goffman’s (1979) extensive content analysis study has been further developed in semiotic categories as an analytical method for ‘reading images’ (Kress and Van Leeuwen 2006; Leeuwen and Jewitt 2001), and Goffman’s work has been continuously revitalised by feminist scholarship for visual analysis (Mager and Helgeson 2011; Zotos and Tsichla 2014; Döring et al. 2016). Consequently, the dimensions originally proposed by Goffman (1979) are now recognised as codes of visual content analysis: (1) *the feminine touch*, (2) *the ritualization of subordination*, (3) *framing* and (4) *licensed withdrawal*; these aspects will be discussed further during the analysis process. To illustrate Williams’s re – shaping of said codes, the analysis incorporates both visual images of Williams, and texts (such as ‘tag – lines,’ Twitter responses and newspaper articles), which accompany them. There is also reference to several other female tennis athletes. Taking this into account, the data set for this project is:

- *One:* The visual images released by the WTA campaign.
- *Two:* The visual image of Serena Williams on the cover of *Sports Illustrated* 2015 (SI), and the Twitter activity surrounding it. This image has been specifically selected for two reasons. One, since the magazine’s launch in 1954, Williams is only the third unaccompanied woman to appear on the cover and, two, because 47% of SI readers had thought that, American Pharoah, the thoroughbred racehorse who won the hallowed American Triple Crown series and the Breeder’s Cup title should have been awarded the accolade in place of Williams. It is also important to note that SI were explicit about the fact that Williams was in control of the photoshoot: @SInow ‘The cover was Serena’s idea, to express her own ideal of femininity, strength and power.’ To gauge fan reaction, the Serena Williams’s Twitter page (@serenawilliams) was selected. Content was scraped – process of extracting specific data from a web page – using Capture for Nvivo. The gathered data were appropriately anonymised, removing author and their Twitter handle. Moreover, to highlight Willimas’s re – shaping, there is a comparative analysis between Williams’s SI cover and Chris Evert’s, the only other female tennis professional to feature on an SI cover and attain the accolade of Sports Woman of the year, cover. The Twitter and visual coverage during the 2018 French Open is also a part of the analysis process, where Williams donned a black cat – suit, which was banned midway by the tournament organisers as it was deemed ‘disrespectful’ and inappropriate’ towards tennis as a sport and the spectators. The final image (Fig. 3) analysed encompasses Williams’s response to such scrutiny.

## Analysis

### Romanticised Pictorial Discourses: Caught in a Bad Romance

In this section, first, I unpick the intentional and unintentional impressions female athletes convey through pictorial representations. Second, applying interview data of some of the athletes photographed in for the WTA campaign, I explore whether their rhetoric is in harmony with their visual representations; in other words, whether they are architecturally unified.

Pre – 2004, women’s tennis had the highest percentage of televised women’s sports (43%), by 2014 this figure had decreased to 6.4% of women’s sports coverage (Cooky et al. 2015). Hence, the purpose of the ‘Strong is Beautiful’ campaign was to ‘develop a closer relationship with fans and attract a new generation of fans to women’s tennis,’ and the personal narratives were to be ‘inspirational.’ The ‘unique combination of athleticism strength and determination on the court and success, interests and *inner beauty off the court* is what makes women’s tennis so attractive to millions around the world’ (Stacey Allaster, Chairwoman and CEO of the WTA, quoted on WTA Tennis website, *emphasis added*).

**Fig. 1:**
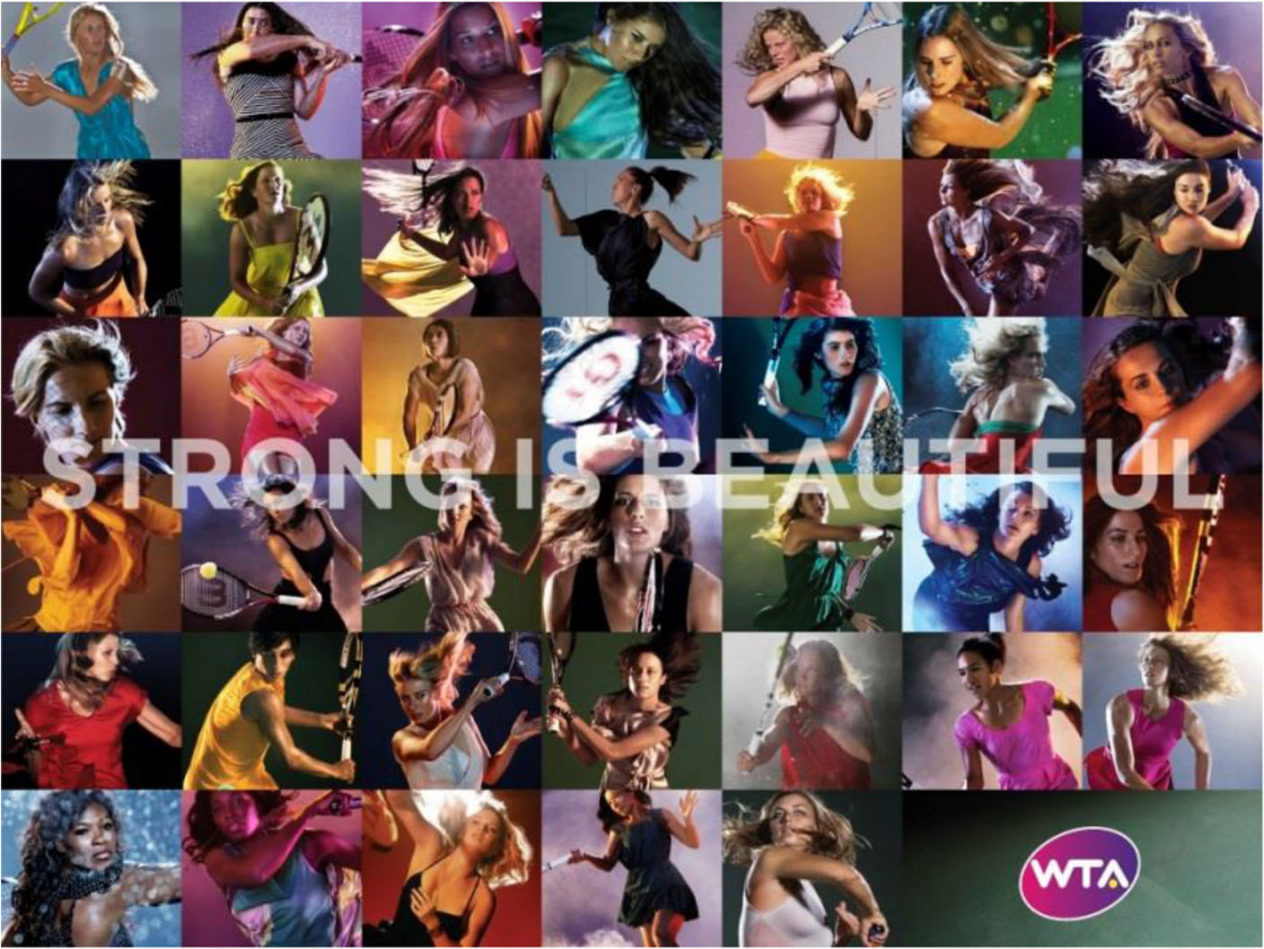
Pictures from the 2011 and 2014 campaign

The message of the campaign itself, however, is questionable. They wrestle with the problem of gender inequality and body positivity but are themselves perpetuating said disputed norms. Rising, first, out of their method of promotion: romanticised pictorial discourse. Figure 1, for instance, exemplifies how the athletes are depicted as forms of picturesque figures. Rather than being perceived as symbols of embodied athletic power, what we have are depictions of mythography. They do not encapsulate the ‘real’ situation, but rather depict an ‘idiom’ of hegemonic femininity, ‘arranged’ and tailored (Goffman 1979: 14). What the viewer perceives is heteronormative, ‘hyper – ritualization,’ and symbolic infantalization of real women in pictorial media representation; particularly the categories of ‘ritualization of subordination’ and ‘licensed withdrawal’ (Goffman 1979). The former, depicts women in inferior positions and poses, the latter entails women gazing into the distance, or as being altogether preoccupied and unaware of the audience’s gaze. The collections of visual images in Fig. 1 portray the latter, consequently, proposing a voyeuristic ‘offer’. A situation where the subject averts their gaze and the viewer is invited to participate as an ‘invisible onlooker’ reducing them to ‘items of information, objects of contemplation, impersonally, as though they were specimens in a display case’ (Kress and Van Leeuwen 2006: 124). All but two of the athletes showcased gaze away, ‘vacantly,’ from the camera lens. Similarly, the camera framing from which we can voyeuristically view each athlete is saliently angled: their heads / bodies are canting and, for some, their mouth as non – smiling and open; all highly objectified patterns of sexuality. There are, for example, varying interpretations of the non – smiling open mouth (Horstmann et al. 2012). Recent research in advertising, however, notes the prominence of this particular pose at the beginning of the twenty – first century, which alongside other signifiers was interpreted as both withdrawal and active sexuality (Hatton and Trautner 2011, 2013; Kuipers et al. 2017). This, in turn, not only overshadows the ethos of the campaign, but also constructs the athletes within a discourse of architectonic otherness. This sense of detachment is the case for both the players and the audience, caused by the athletes’ poses, which are not necessarily athletic, and their displacement from the sport’s ‘natural habitat,’ the court. What they depict is the civilisation of their Grotesque athletic bodies into defined and immutable Classical bodies. This rearrangement is noteworthy as women who opted to foreground their athletic identities preferred to be photographed in the location of their sport and in their athletic attire (Barak et al. 2018). Stripping the female athletes in Fig. 1 of both factors not only mediates them as an Other in terms of gender, in relation to their male counterparts, but also an Other to their ontological embodied selves. Specifically, the message is ambivalent and does not reflect their ontological essence of being athletes *or* women, and the individuality of the subject is not central. Although the message in Fig. 1 does not centralise sexuality, nonetheless, what we have is an idealisation of the female figure. What we view is a visual romanticised representation, arguably, an epitome of mythic ideal femininity, rather than a display of the physicality, embodiment, and earthiness of being a female *and* an athlete. As delineated below, this fragmentation becomes increasingly sharper as one begins to analyse interview rhetoric, which discloses a discrepancy between the attitude advocated by the picturesque images produced by the campaign and the actual voices of the female athletes.

The media coverage of the 2015 Wimbledon final between – between Williams and Garbiñe Muguruza is a revealing one. First, despite the WTA’s ‘Strong Is Beautiful’ tagline the piece, written by Ben Rothenberg (2015), re – ignites the question of sports and beauty, implying that the movement had been unsuccessful in conveying their message. Second, it further unravels architectonic struggle experienced by female tennis plays when it comes to negotiating ambition, their physicality, and femininity. Rothenberg (2015) notes that ‘[Williams] has large biceps and a mold-breaking muscular frame, which packs the power and athleticism that have dominated women’s tennis for years. Her rivals could try to emulate her physique, but most of them choose not to.’ Their decision, it would seem, is partially driven by their coaches, who are more often than not also male. In a build – up interview, Tomasz Wiktorowski, the coach of Agnieszka Radwanska stated that: ‘It’s our decision to keep her as the smallest player in the top 10. Because, first of all she’s a woman, and she wants to be a woman.’ When questioned about the statement, to a certain degree, Radwanska seems to agree with her coach’s position: ‘Of course I care about that as well, because I’m a girl … But I also have the genes where I don’t know what I have to do to get bigger, because it’s just not going anywhere.’ Other players evidently also remain in a complex paradox of social discourses around femininity and the ambition to excel. To the extent of expressing distress at viewing unadulterated and ‘candid’ (Goffman 1979: 14) images of them in ‘live action.’ The quote below, for example, is a response by Andrea Petkovic (then ranked 14th) when questioned about female athletes and body positivity. Petkovic confessed discomfort with viewing images of herself while hitting a two – handed backhand, when her arms appeared must ‘bulging’:

I just feel *unfeminine*. I don't know — it's probably that *I'm self-conscious about what people might say*. It's stupid, but it's insecurities that every woman has, I think. I definitely have them and I'm not ashamed to admit it. I would love to be a confident player that is proud of her body … People say, ‘Oh *you're so skinny*, I always thought you were huge.’ And then I feel like there are 80 million people in Germany who think *I'm a bodybuilder*. Then, when they see me in person, they think I'm O.K. **(**Rothenberg 2015, *emphasis added*).

Petkovic’s body commentary is strikingly counterproductive to the pictorial discourse championed by the WTA’s campaign, which both Radwanska and Petkovic advocated. Petkovic readily condemns the explicit social expectations of femininity, more importantly, she reveals ‘insecurities’ with her ‘physicality’, which leaves her ‘self – conscious.’ Her rhetoric, in reference to women and body-policing in general, ‘Women, when we grow up we’ve been judged more, our physicality is judged more, and it makes us self-conscious,’ signifies that ontological fragmentation and architectonic otherness begins to emerge at an informative age. Importantly, said insecurities permeate into adulthood and professional choices, as discussed earlier. The fundamental configuration required – between the visual and verbal – for attaining architectonic wholeness is absent. Therefore, contrary to women in popular media Krawitz (2014), for professional female athletes, in terms of body and beauty, ‘live play’ ensures complete transparency. They cannot veil themselves behind re – touched images or ‘reel – life.’

The ‘Strong is Beautiful’ images do not replicate the athletes’ lived embodied experience as high performing athletes. More specifically, there is no sweat and little signs of struggle, strain, and exertion on their expressions. It also reveals how audiences, maybe unintentionally, tailor or misconstrue the players’ actuality. When individuals meet Petkovic in person, for example, they openly express their shock at her ‘skinny [ness].’ Disclosing, therefore, not only the process of how fans’ construction of female athletes’ selfhood is constrained through hegemonic discourse, but also how said discourses are reinforced: displacing aspects such as competitiveness, aggression and strength during live action renders female athletes as masculine and ‘huge.’ The language from the excerpt above implies that external factors, such as audience commentary on her physicality, have a complex impact on both, Petkovic’s emerging sense of herself as feminine and a female athlete. The fact that female athletes are aware and are not ‘confident’ enough to be ‘proud of [their] body,’ implies architectonic abrasion. This fragmentation of their selfhood becomes increasingly complex as they strive to negotiate and maintain a feminine persona through popular media photographs. They are unable to attain wholeness by emulating the visual aesthetic displays of their powerful bodies, with equally powerful rhetoric. For desire of approval, and to subscribe to social discourses of femininity, there is an eclipsing of the campaign’s slogan of strong, indeed, being beautiful, hence, rendering it as an illusion. Viewed collectively, the photographs in Fig. 1 can be interpreted as artistic representations of the subject. A mechanical unity where ‘Art is too self – confident, audaciously self – confident, and too high – flown, for it is in no way bound to answer for life. And, of course, life has no hope of ever catching up with art of this kind’ (Bakhtin 1990: 01). Put in context, the WTA visuals are an *ethereal* and ‘other world’ representation which the female athletes, understandably, are unable to integrate into their identities, therefore, there is an absence of architectonic unity. Indeed, from within, one can only affirm their physical existence (‘self – sensation’), but we experience our outward aesthetic appearance in relation to the world around us (Bakthin 1990). This is not to say that one cannot visualise oneself through self – observation and imagination. Imagine peering into a mirror, if you will, and self – sensing that your facial features are aesthetically pleasing. In order to authenticate your perusal of yourself, however, and ‘make it part of a concretely viewable [architectonic] whole’ you must re – construct your architectonic by factoring in how your outward image emerges ‘*out* of the other and *for* the other’ (Bakhtin 1990: 30, *original emphasise*). From a Bakhtinian perspective, female athletes such as Petkovic may self – sense their bodies as strong and powerful yet feminine, however, for architectonic unity it is essential for this self – sensation to be vivified by others, without that her inner image is ‘detached’ and ‘empty’ (Bakhtin 1990).

At this point, it is also essential to acknowledge another facet of unauthentic digital self – presentation. Around the launch of the campaign, Maria Sharapova, also represented in Fig. 1, proclaimed that, ‘I always want to be skinnier with less cellulite. I think that’s every girl’s wish.’ Arguably, Sharapova’s words also suggest a mechanical architectonic wholeness. Alternatively, there is, of course, the argument that certain female athletes consciously self – monitor and perpetuate gender performances due to neoliberalist economic drivers. Feminist research has explored prominent social discursive tendencies where, at times, skill and talent have been overshadowed by sexual attractiveness and physicality. Konjer et al. (2019) coin this as ‘erotic capital.’ The difference between Sharapova’s and Williams’s off – court endorsements and earnings, in comparison to their on – court achievements and earnings paints a telling picture. The London School of Marketing’s (2015) marketability list only recorded two women in the top 20: Williams (20) and Sharapova (12). Williams earned half the amount, in comparison to Sharapova, despite holding a 16 – 1 winning record against her (Rawlinson 2015).

Arguably, the intensity at which Architectonic Fragmentation is experienced is not unifiable. Experience may be determined by the level of media attention a certain female athlete receives and how strongly they identify with their athletic self. The emergence of economic incentives (Konjer et al. 2019), from within the sport further reaffirms social pressures for female athletes to be simultaneously both, ‘pretty and powerful’ (Bruce 2016: 01). This in turn perpetuates the social practice of exaggerating sexual attractiveness and trivialising and undervaluing their athletic achievements – a discourse long scrutinised and criticised by traditional feminist scholarship (Duncan and Hasbrook 1988).

For marketability, however, being ‘pretty’ is not enough. Femininity and playing to traditional gender discourses, is also essential both on and off the court (Smith and Sanderson 2015). The tension – filled relationship between sports journalists, predominantly male, and Victoria Azarenka is a perfect reflection of this (Jenkins 2013; Hinds 2013). In a post – feminist culture that advocates self – confidence and individual resilience, criticism of Azarenka’s on – court attitude implies that women, especially visible women, are caught in yet another inconsistency. Gill and Kanai (2018) note that, ‘pure self confidence when performed … often does not pass the test of “likeability”’ (Gill and Kanai 2018: 322). For a female athlete to be likeable, she must be relatable and display some form of feminine insecurities and vulnerability when, in fact, elite female athletes are anything but ‘normal.’ Perhaps this performance of feminised gendered discourses is what makes certain female athletes more lucrative than others. Athletes are highly dependent on their public image, which is a significant factor in obtaining neoliberalist endorsements. Historically, Williams’s statements regarding female athletes and body image have been ambiguous. She often projects her physicality in relation to fitness, rather than desiring it to be ‘skinny.’ Perhaps, therefore, sports journalists were, and are still able, to connotate ‘large biceps’ and a ‘muscular frame,’ as ‘mold – breaking’ for high performing female athletes. When requested to comment on the popular media’s relentless scrutiny of her physique, she responded: ‘I don’t have time to be brought down, I’ve got too many things to do. I have Grand Slams to win, I have people to inspire, and that’s what I’m here for’ (Wells 2015). The change in Williams’s visual images and direct rhetoric, post – 2014, however, allude otherwise.

### Bodies of Power: Myth – Breaking and Myth – Making

In 2015 Williams was also named the Sports *Person* of the Year by (SI).

The cover itself created a flurry of response on Twitter, with audiences accusing SI of altering Williams’s image for it to appear sexy. SI promptly announced that ‘The cover was Serena’s idea, to express her own ideal of femininity, strength and power’ (@SInow). How does, then, Williams’s individual discourse of femininity, differ from the WTA’s collective pictorial promotion of gender equality? Below, utilising the aforementioned visual content analysis codes, I argue how Williams’s 2015 cover can be perceived as ‘myth – breaking,’ in the sense that it overrides several of the overarching gender stereotypes associated with women in commercial pictures. In its place, what we begin to witness are the embryonic stages of ‘myth – making’, a process in which Williams elevates iconology beyond gender boundaries and into realms of becoming a myth – like feminist symbol and in the process overriding architectonic fragmentation. ‘Myth – like’, here, refers to a transcendental form of embodiment, where here gendered body is not the focal point, but rather as an allegorical expression of power.

The analysis of Fig. 1 has revealed that when a subject is ‘in art’ they ‘are not in life’ and for one to achieve unity between the architectonic constituent elements of selfhood one must have the capacity to be answerable, termed as ‘answerability’ (Bakhtin 1990). From a Bakhtinian perspective ‘answerability’ encapsulates the mutual relationship between the art, the artist, and the viewer. An artist, for instance, is the ‘active form-giving energy’, which manifests in his ‘durably valid cultural product’: his art / his subject (Bakhtin 1990: 08). For said subject to become ‘whole’ and hold meaning, the viewer must be able to understand the art within their own ‘everyday’ experience. In the case of this paper, I will take the liberty of appropriating ‘answerability’ a little differently. For Williams to extricate herself from discourses surrounding female athletes and to generate speaking, rather than silenced pictures, it is pivotal for the her to personally engage in the aesthetic process. This ‘engagement’ does not simply refer to Williams choosing how she wished to be portrayed on the SI cover, but also refers to the final visual representation having the capacity to *engage* with its viewer(s) and have the ability to *answer* any impeding social reactions.

**Fig. 2:**
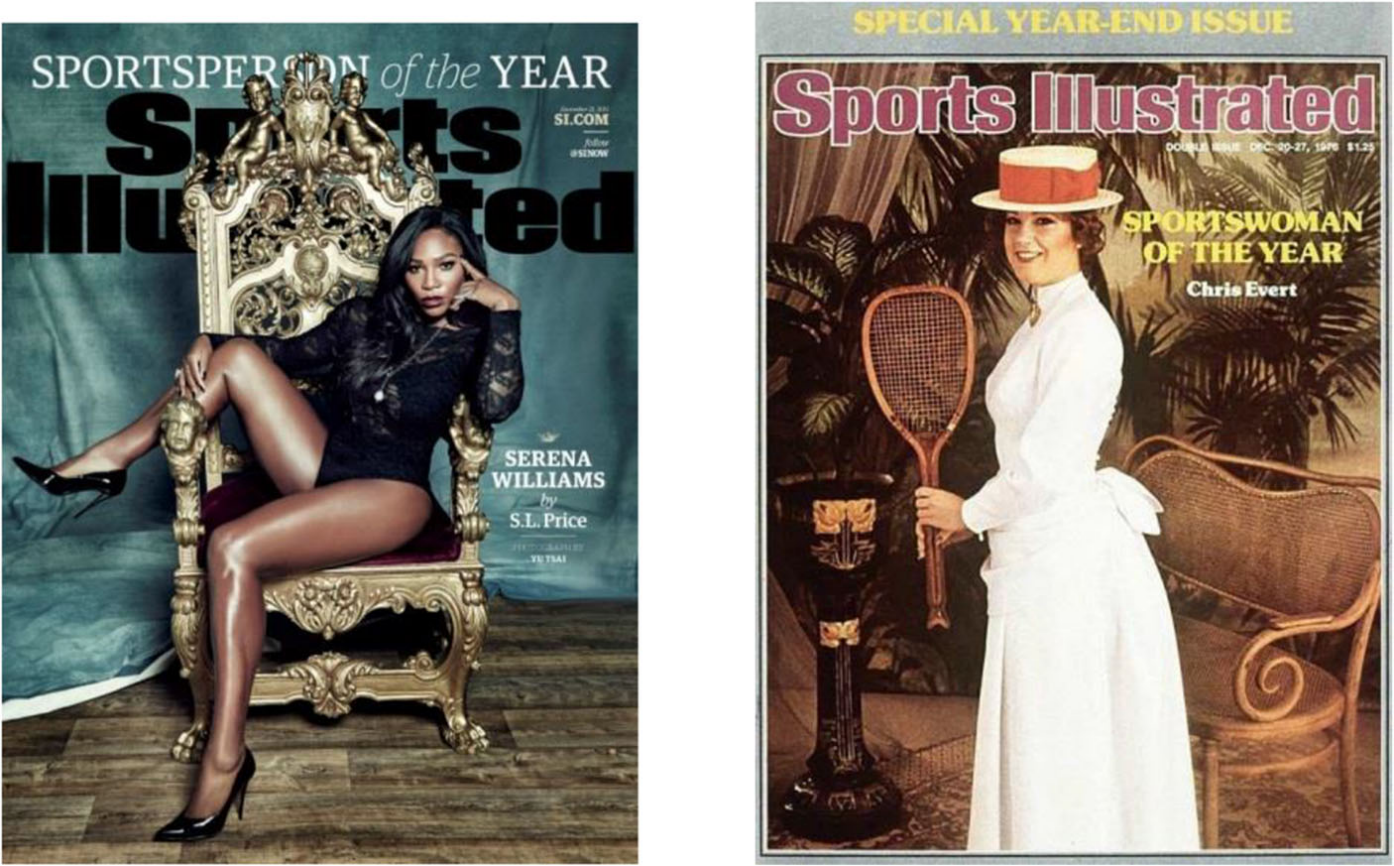
*Sports Illustrated* (2015) and Sports Illustrated (1976)

Williams’s image in Fig. 2 performs to none of the representations of hegemonic femininity visible in Fig. 1 and Evert’s 1976 cover. When considered as a whole, the 1976 Sports *Woman* of the year cover depicts all the gendered hallmarks identified by Goffman (1979). Although Evert does make direct eye contact with the audience, and smiles, her facial expression is somewhat ambiguous, if not vacant. Research developing on Goffman, (Williamson 1978; Rodero et al. 2013), argues that women in advertising have a dual functionality, as both representations (of the product being sold) and objects themselves. The messages of objectification are created through a technique known as ‘framed distance,’ exercised to build a narrative and contextualise an image (Goffman 1979). There are three levels of distance: the intimate personal distance, the social distance, and the public distance. Applying the public distance approach, where the subject is shown in full – shot with space around them, the narrative created for Evert is one of femininity, leisure, and the private home sphere. Considering that Evert had won the accolade of sports*woman* of the year she has not only been located within a home setting, but it is also questionable why she has been attired in dresses worn by female tennis plays between the 1880s and 1920s, a time period where female tennis players competed as amateurs and were unpaid.

Along with the 'male – gaze,’ in his tools for visual analysis, Goffman (1979) also applied the concept of ‘the feminine touch,’ where women are often depicted as lightly touching or caressing objects. Alternately, men are generally shown as *purposefully* grasping objects, which coincides with how Evert is clasping her tennis racket at an awkwardly purposeless right angle. Goffman (1979) argues that ‘females … are frequently posing while using their fingers and hands to trace the outlines of an object, or to cradle it or to caress its surface’, women are also shown, he continues, touching [a part of] themselves’ (Goffman 1979: 29). Typically, this form of touch encourages the notion that the subject is sexually available, weak, and vulnerable in relation to men (Goffman 1979). The visual representation of Evert, simultaneously conceals, if not trivialises, her athletic achievements and fulfils the femininity ideal desired by men of the period (Mulvey 1989); she is portrayed from a male gaze perspective, for the' male – gaze' perspective.

The same, however, is not the case for the 2014 cover. Rather than gazing away from the camera, which is a reoccurring characteristic in Fig. 1 and the 1976 cover, Williams meets the gaze of her viewer directly. Meeting the viewers gaze head – on, mouth remaining closed, is often interpreted as a direct ‘demand’ or challenge, and the characteristic of the ‘demand’ is dependent on the subject’s overall facial expression and bodily posture (Goffman 1979). Coupled with the fact that Williams is photographed *en face* (frontal image with both sides of nose visible) and direct gaze, her expression can be interpreted as a challenge. Although the *en face* camera angle is typically associated with masculinity, this is not to say that Williams is striving to project a masculine persona (Kress and van Leeuwen 2006; Mager and Helgeson 2011). Considering the controversy surrounding the cover, a dare, perhaps, for the viewer to question her place not only as the rightful champion to sit on the figurative throne of Sports *Person* of the year, but also the actual throne depicted on the cover. In contrast to the photographs in Fig. 1 and the 1976 cover, Williams’s hand placement, and the fact that she is not holding a tennis racket, is an essential element in the myth – making process for two reasons. First, although placed upon her person, her hands are empty, and their positioning is noteworthy. Opposing the Goffmanian notion of ‘the feminine touch,’ and ‘knee bend,’ they are not caressingly placed upon a vulnerable location on her person, such as the neck and chest, but positioned authoritatively at the side of her face and on her bent knee, flung over an armrest. She reclaims two characteristics associated with vulnerable femininity and re – constructs them into a discourse of empowerment. Second, there is a particularly interesting audience response in Table 1, which bemoans the fact that the SI cover does not represent a tennis player. Eliminating the racket has functioned as a strategic choice in Williams re – shaping her image as one beyond tennis. For instance, it creates a persona that does not rely on her tennis, or biological femininity, recognising her as neither a tennis player nor female, but as a ruler. The ceremonial throne of Sports *Person* of the Year immediately establishes the ruling status of whoever occupies it. It places prestige and power and, arguably, Williams deliberately benefits from its emblematic quality.

**Table 1:** Twitter Response to 2015 *Sports Illustrated* cover

They photoshopped the **** out of #SerenaWilliams’ thighs for that #SportsIllustrated cover. Geez.	
Wait did @serenawilliams lose weight or did @SInow photoshop her cuz I don’t ever recall them thighs ever being that small	
I don’t like that cover photo of Serena Williams on SI because they made her thighs look nonexistent.	
@serenawilliams Yaaaaasssss hontea! You are slaying on this cover! Your thighs look slimmer? Hope they didn’t photoshop your thunder.	
Sports illustrated didn’t do justice to Serena’s thighs but Damn look at @serenawilliams ankles	
Photoshopped #SerenaWilliams until she looked slim. Beautiful picture but she better with her normal body.	
Strong, confident, powerful...there was no need to photoshop her body! #SerenaWilliams #SISportsperson	
@SInow brilliant that @serenawilliams is your #SportspersonOfTheYear but the Photoshop is undermining.	
I LOVE @serenawilliams but there’s too much photoshop in the SI cover, especially on her face.	
@SInow Really? @serenawilliams. You are worthy of more than an SI Swimsuit cover. Represent feminine, strong and powerful TENNIS PLAYERS!	

Moreover, in comparison to the 1976 cover, the textual content is kept to a minimum and noticeably the writing is placed on the right bottom corner, which is the exact place where the conventional direction of reading ends. Western occidental reading habit follow a zigzagging pattern, that is the reading direction followed is from the left top to the right top and then down to the left bottom ending at the right bottom (Afsari et al. 2016). The distribution of the visual and written content at the ‘end point’ not only increases the effectiveness of the message, but it also ensures that the visual image takes precedence over the written message that simply reads ‘Serena Williams.’ Her person as ruler is further reiterated by the intimate distance framing narrative (Goffman 1979; Kress and Van Leeuwen 2006). Williams’s image is at the centre and forefront of the cover and occupies the majority of the space available, to the extent that the ‘Sports Illustrated’ and ‘Sportsperson of the Year’ remain in the background. The terminology is also essential. Williams is not the Sports *Woman* of the year, but the Sports *Person.* The viewer is not offered an opportunity to build any alternative narrative around the image, other than one of ruler. A persona that has provided the ignition for Williams attaining architectonic wholeness, by offering paradigms of re – shaping that enable her to transcend the existing schemas around female athletes and femininity.

### Outward Images and Rhetorical Alignment

Previously, I argued that female tennis players often encounter Architectonic Fragmentation because they are unable to achieve ‘meaning’ between their projected images of empowerment and their rhetoric, therefore, classifying them as ‘plastic and pictorial’ (Bakhtin 1990: 28). This, however, is not the case for Williams. The following section discusses her rhetoric and the manner in which she chooses to visually present herself are collective and dynamically coherent. During the 2018 French Open championships, Williams wore a catsuit, which was eventually banned as it was deemed ‘inappropriate and disrespectful to the game’. When questioned about her outfit choice Williams’s response continued to reinforce and maintain the self – shaping that began with the 2015 SI cover:

I feel like a warrior in it, like a warrior princess kind of, [a] queen from Wakanda … I’m always living in a fantasy world. I always wanted to be a superhero, and it’s kind of my way of being a superhero. I feel like a superhero when I wear it (Williams, quoted in Barr 2018).

**Fig. 3:**
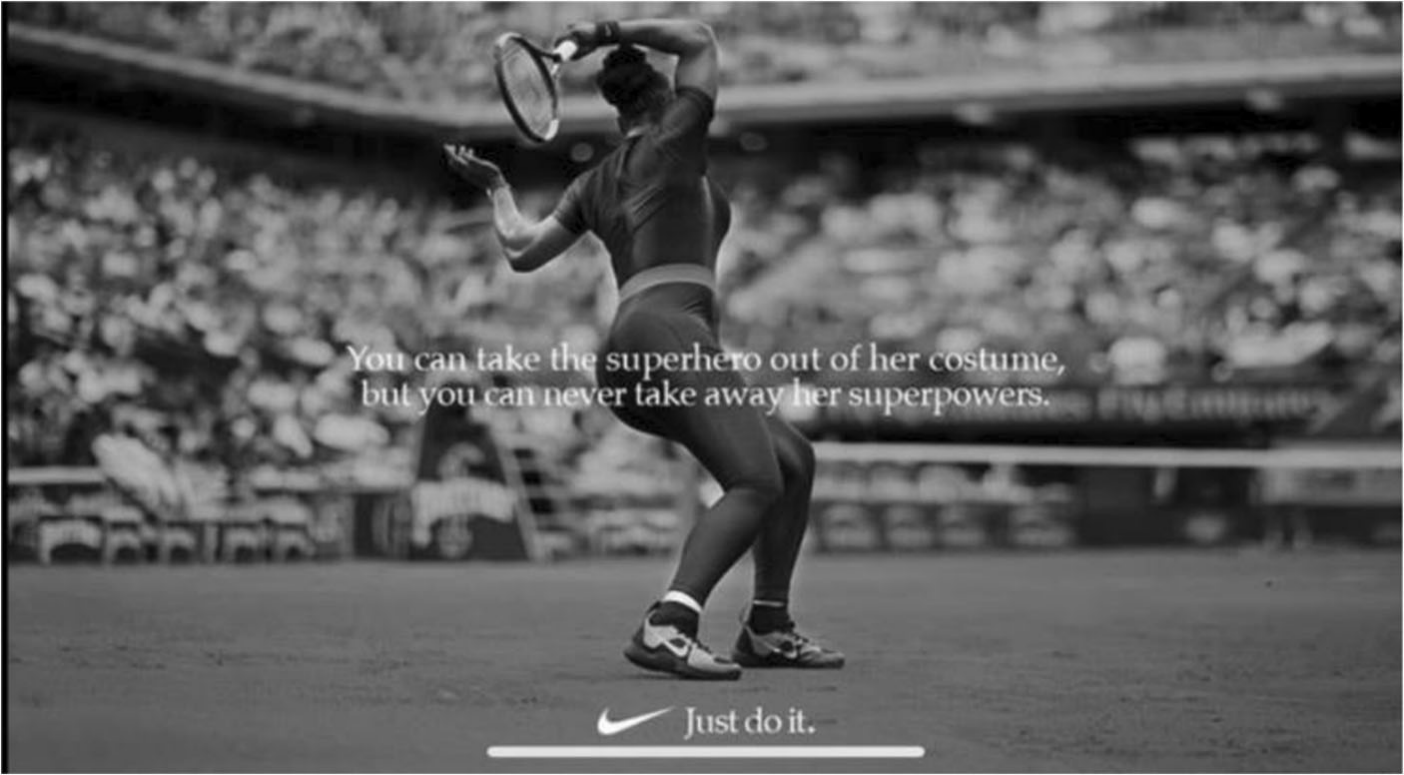
Nike’s response to *French Open catsuit* ban (2018)

There are two noteworthy aspects in Williams’s response: the language she uses to describe herself and that the language remains gendered. The words ‘warrior princess’, ‘queen’ and ‘superhero’, for instance, not only project images of power, royalty, and enchantment, but also detangle Williams from femininity discourses associated with female athletes. Her gender is not the main promotion; her exalted, almost mythical status is. This is further reinforced by Williams’s referral to Wakanda, a fictional location in the Marvel comic universe. Following the French Open Nike published the image displayed in Fig. 3. The image indeed displays power and strength, depicting Williams in ‘full – flight’, in the middle of a match, in that catsuit. The slogan that accompanies the image, however, is just as empowering and a pivotal element in maintaining Architectonic Wholeness: ‘You can take the superhero out of her costume, but you can never take away her superpowers.’ She does not apologise for either, her cultivated self – representation as a ‘superhero’ and her outfit. Williams, therefore, manages to project internal and ‘*external* unity and continuity’ (Bakhtin 1990: 35). Upholding her re – shaped image, for the 2019 French Open, Williams had the following worded intertwined into her outfit: ‘Mother,’ ‘Champion’, ‘Queen’ and ‘Goddess.’

It is important to acknowledge that achieving architectonic wholeness is not a solitary process. Indeed, the outward persona can only endure ‘if the other [or viewer also] did not create it: aesthetic memory is *productive* – it gives birth, for the first time, to the *outward* human being on a new plane of being’ (Bakhtin 1990: 36, *original emphasis*). In Williams’s case, she is recreated as a divine mythical queen. The Twitter responses, in Table 2, are indicative of how her re – shaping and self - representation has permeated the viewers’ rhetoric. In this sense, both the viewer (audience) and the art (Williams) are simultaneously appropriating, exploiting and undermining social discourses around feminism, femininity, and female athletes.

**Table 2:**
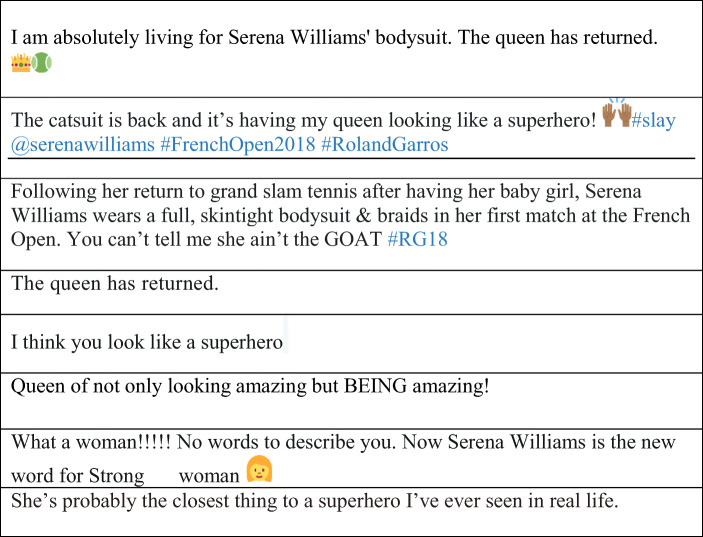
Twitter Response to 2018 *French Open Catsuit*

## Conclusions

Applying Bakhtin (1990) architectonics, considering feminist scholarship concerning media platforms, and heteronormative discourses surrounding the body, this article intended to explore the embodied experience of female athletes, specifically female tennis players. A review of the current literature disclosed an interesting feminine ‘civilizing process’ of the athletic female body within media and pictorial depictions, not only for marketability, but also for compliance to social conventions on femininity. Indeed, some female athletes often utilise personal social media platforms for self – shaping and self – representation (Smith and Sanderson 2015; Toffoletti and Thorpe 2018a), in order to align with heteronormative discourses.

For example, a visual content analysis of images from WTA’s ‘Strong is Beautiful’ campaign, designed to promote body diversity and positivity, depicted a devolved representation of the female tennis plays featured in the photographs. The pictorial representations, for instance, reverted to a subjugating ‘civilizing processes,’ that resulted in detaching and Othering them from their embodied sense of self as athletes. This dichotomy was further reiterated in the interview rhetoric of several players, which implied Architectonic Fragmentation as they struggled to find an equilibrium between their athletic embodiment and conventional femininity . Particularly if they are unable to attain architectonic wholeness between their *authentic embodied self* and their romanticised *marketable image*. An image situated around the ‘male – gaze.’

However, through a content analysis of Williams’s 2014 SI cover, her response (Fig. 3) to being banned from wearing an ‘inappropriate’ catsuit during ‘live play,’ and her interview rhetoric, this paper argued that Williams is paving an alternative path for attaining architectonic wholeness: by conscious self re – shaping of her pictorial depictions, and , more importantly, supplementing them with rhetoric that aligns with the projected imagery. In doing so, she not only begins to re – configure Western perceptions of femininity, but also foregrounds the *multiplicity* of embodied femininity. In other words, embodiment of ‘femininity’ is polysemic and, akin to the Bakhtin (1990) author – hero relationship; this is fortified by the audience’s response in Table 2. It is essential to note that, for attaining architectonic Wholeness, Williams does not re – construct her image as androgynous and ambiguous in gender, to accommodate traditional feminine athletic characteristics, such grace balance, and aesthetics (Crossman et al. 2007). Rather, her version of femininityacknowledges both, her ‘femaleness’ and her powerful physical strength. Therefore, challenging historical media constructions of women in sports where ‘women play only a subordinate and/or sexualised role’ (Bernstein 2002: 426), and are commodified for the ‘male – gaze.’ Williams’s SI cover, contrastingly, imposes an alternative interpretation of empowered femininity, one re – shaped for the ‘female – gaze,’
